# Altered Scale-Free Dynamics of Spontaneous Brain Activity in Post-Traumatic Stress Disorder: Relationship to Impaired Functional Connectivity

**DOI:** 10.3390/brainsci16080827

**Published:** 2026-08-04

**Authors:** Mihai Popescu, Elena Anda Popescu, Thomas J. DeGraba, John D. Hughes

**Affiliations:** 1 National Intrepid Center of Excellence, Walter Reed National Military Medical Center, Bethesda, MD 20889, USA; 2 Psychological Health Branch, Walter Reed Army Institute of Research, 503 Robert Grant Ave, Silver Spring, MD 20910, USA

**Keywords:** post-traumatic stress disorder, magnetoencephalography, detrended fluctuation analysis, scale-free dynamics, functional connectivity

## Abstract

**Highlights:**

**What are the main findings?**

- Post-traumatic stress disorder is associated with alterations in the scale-free dynamics of resting-state cortical activity characterized by lower DFA exponents of beta-band oscillations.
- These alterations in local scale-free activity strongly correlate with reduced inter-hemispheric functional coupling.

**What are the implications of the main findings?**

- These findings suggest that the reduced resting-state inter-hemispheric functional connectivity and altered local scale-free activity are inter-connected hallmark features of a breakdown of the spatiotemporal brain dynamics in PTSD.
- Our results suggest that therapies with the potential to normalize local scale-free brain activity may also contribute to the restoration of functional brain connectivity in PTSD.

**Abstract:**

**Objective:** Post-traumatic stress disorder (PTSD) has been linked to reduced functional brain connectivity, but it is unclear if altered local network properties contribute to these disruptions in functional coupling. Computational studies have demonstrated that dynamical states characterized by scale-free local activity optimize the long-range functional coupling in neuronal networks. Our study uses resting-state magnetoencephalographic recordings to investigate if characteristics of scale-free dynamics of local cortical activity are associated with alterations in long-range functional connectivity in PTSD. **Methods:** Participants (*n* = 96) were service members with combat exposure and various levels of post-traumatic stress severity (PTSS). We estimated the amplitude envelopes of beta-band cortical activity in 134 cortical regions and we characterized their scale-free dynamics using the power-law scaling exponent determined by detrended fluctuation analysis. We assessed the correlation between PTSS, scaling exponents, and functional coupling between homologous anatomical areas of the two hemispheres. **Results:** We found negative correlations between PTSS and scaling exponents as well as between PTSS and inter-hemispheric coupling (IHC) predominantly in prefrontal and temporal regions. Positive correlations between scaling exponents and IHC were present in a large number of regions, with high correlations across primary and unimodal association cortices and lower correlations across high-order heteromodal processing areas. **Conclusions:** Our findings suggest that mechanisms that regulate scale-free dynamics of local cortical activity may contribute to alterations of long-range functional connectivity in PTSD. **Significance:** Therapies with the potential to normalize local scale-free brain activity may lead to the restoration of functional brain connectivity, offering a promising avenue for PTSD treatment.

## 1. Introduction

Post-traumatic stress disorder (PTSD) is a chronic condition that can develop after exposure to psychological trauma. The lifetime prevalence of PTSD in the US population is estimated at 6–7% [1,2,3], but higher rates are present in high-risk groups such as individuals exposed to military combat [4,5,6] or service members and civilians with a history of traumatic brain injury [7,8,9,10,11,12]. Previous research studies suggest a complex multifactorial pathogenesis of this disorder with alterations manifesting at multiple levels of brain organization. 

Accumulating evidence suggests that brain function relies on the dynamic integration and segregation of information processing in distributed networks and is determined by the coordinated activity of ensembles of neurons bound together over local and global spatial scales by synchronization of neuronal oscillations of various frequencies. Dynamic changes in phase and amplitude coupling of neuronal oscillations result in ensembles of neurons having a wide range of sizes and lifetimes that form and dissolve transiently to generate a large repertoire of functional states, which is a fundamental characteristic of a self-organized critical state in neuronal networks [13,14]. One hallmark of the critical state is the spatiotemporal dynamics of the local network activity with statistical properties characterized by power-law scaling and the presence of long-range temporal correlations (LRTCs) reflected in fluctuations over a wide range of temporal scales [15]. Power-law scaling has been independently observed using different measures of neuronal networks activity, such as the distribution of neuronal avalanches recorded in cortical slices or in vivo [13,16] or the decay of temporal correlations of the amplitude envelope (AE) of spontaneous brain oscillatory activity recorded noninvasively with EEG or MEG [15,17]. Computational studies have shown that these characteristics can jointly emerge in neuronal networks in a self-organized critical state for a particular range of excitatory to inhibitory connections [18]. 

Previous computational studies have also shown that the scale-free, power-law behavior of the infraslow fluctuations in amplitude envelopes of local neuronal oscillatory activity optimizes the long-range functional coupling in neuronal networks [19], i.e., when networks are in a critical state they are most responsive to inputs from distant regions and more effective at influencing the activity in distant regions. Alterations in the dynamics of local brain activity associated with brain disorders, which can be due to changes in the local excitation to inhibition balance, can therefore influence the long-range functional connectivity by limiting the network’s ability to coordinate activity in distant brain regions. Although many studies linked PTSD to widespread alterations in long-range functional connectivity [20,21,22,23,24], the potential relationship between the scale-free dynamics of local brain activity and such disruption in long-range functional coupling has not been explored. Investigating this relationship is important because it can bridge the mechanisms regulating the temporal complexity of local brain activity to large-scale network interactions (functional connectivity), thereby improving our understanding of PTSD pathophysiology and potential treatment strategies. In one of our recent studies that used resting-state MEG recordings, we found that PTSD is associated with lower coupling of infraslow fluctuations of the amplitude envelope across large-scale cortical networks, which was primarily manifested in the beta frequency band [21]. Notably, beta-band activity reflects long-range interactions between cortical regions [25,26,27] underlying cognitive, emotional or sensorimotor states that can occur in the absence of ongoing sensory input [27,28], such as during resting-state recordings with eyes closed that are the focus of our investigation. In this study, we hypothesized that the previously observed lower coupling of the beta-band AEs may reflect in part some alterations in the mechanisms that govern the local scale-free dynamics of the regional infraslow AE fluctuations. To test this hypothesis, we examined the association between post-traumatic stress severity (PTSS), long-range functional connectivity and power-law scaling behavior of the AEs of beta-band activity, using data from the same sample of participants as our previous study.

## 2. Methods

### 2.1. Participants

The sample of participants included in this study has been described in detail in our previous study [21], but some information is included below for convenience. Participants (*n* = 96, all males) were service members enrolled in an outpatient program for patients with post-concussive and post-traumatic psychological health symptoms at the National Intrepid Center of Excellence (NICoE), Walter Reed National Military Medical Center, Bethesda, MD, USA. The study was approved by the Institutional Review Board of the Walter Reed National Military Medical Center in compliance with all applicable federal regulations governing the protection of human subjects. Informed consent was obtained from each participant before participation in the study. 

All participants completed the PTSD Check List-version 5 (PCL-5), a 20-item self-report scale used to screen individuals for PTSS [29,30]. PCL-5 items rate the severity of re-experiencing, avoidance, emotional numbing, negative thoughts, and hyperarousal symptoms elicited by stressful experiences. PCL-5 scores across participants ranged from 2 to 71 with 32 of the participants meeting the DSM-V criteria for PTSD diagnosis. The exposure to combat experiences generally occurred over extended periods of time, during which the participants have also experienced one or more mild traumatic brain injuries (mTBI, defined according to criteria established by the American Congress of Rehabilitation Medicine [31]). Across participants, the time since the last injury was 6.3 ± 4.8 years. As mechanism of injury, 78 participants had both blast and non-blast injuries, 14 participants had non-blast-only injuries, and 4 participants had blast-only injuries. 46 participants had at least one mTBI with loss of consciousness (LOC). PCL-5 scores were not significantly different between the subgroup of participants who had mTBIs without LOC and the subgroup of participants who had mTBIs with LOC (Mann–Whitney test: u_A_ = 1154, z = −0.03, *p* = 0.97).

Participants also completed the AUDIT-C alcohol consumption screening form [32] and the PHQ-9 depression form [33]. Five participants had PHQ-9 scores in the range of severe depression (PHQ-9 scores greater or equal to 20). Depression severity was not an exclusion criterion since a significant proportion of patients with PTSD have depressive symptoms [34], possibly reflecting a trauma-related phenotype that is distinct from major depressive disorder in the absence of trauma exposure [35]. Participants were not excluded from the study based on medication use. Twenty-eight participants were taking antidepressant medication, 11 participants were taking Prazosin and 27 participants were taking Gabapentin for headache prophylaxis. No participants in this study had a history of use or abuse of recreational drugs. No significant correlations were present between PCL-5 scores and age, education, AUDIT-3 scores, and full-scale IQ [21].

### 2.2. MEG Data Acquisition and Processing

MEG data acquisition and pre-processing was performed, as explained in detail in our previous study [21]. We acquired MEG data with a duration of 5 min and 1 kHz sampling rate using the Elekta VectorView™ whole-head MEG system (Elekta-Neuromag Oy, Helsinki, Finland). Participants were instructed to close their eyes, relax and remain awake during the recordings. 

The pre-processing steps included band-pass filtering between 1 Hz and 100 Hz, powerline filtering at 60 Hz, and down-sampling at 500 Hz. Independent Component Analysis using an Infomax algorithm (EEGLAB v4.311, [36]) was used to remove cardiac and eye movement interferences, as well as other sources of external artifacts after visually inspecting their temporal features, frequency spectrum, and spatial mapping in accordance with existing guidelines [37]. The denoised signals were subsequently band-pass filtered in the beta frequency band (13–30 Hz). 

The cortical surface was determined from T1-weighted MR images of each participant using FreeSurfer v5.1.0 [38]. The source reconstruction was done using Brainstorm v3.4 [39]. Cortical currents with unconstrained orientation were estimated at 10,000 locations with a minimum norm estimator (MNE, [40]) using a depth weighting parameter of 0.5 and a multiple overlapping sphere model of the volume conductor [41,42]. The inverse projection operator incorporated a diagonal noise-covariance matrix derived from 1 min long empty room noise recordings filtered in the beta frequency band. 

**Figure 1:**
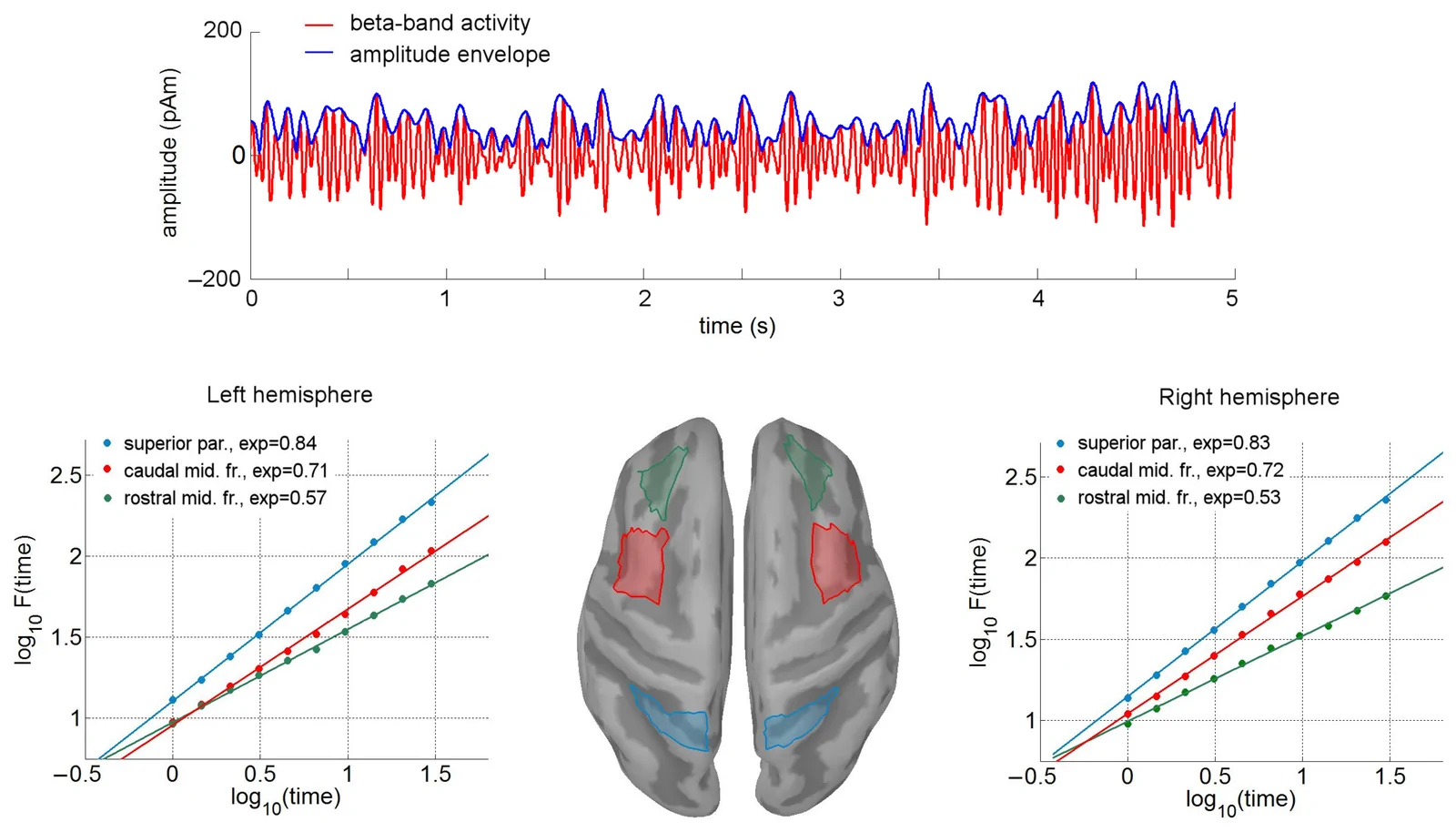
**Upper panel**: Example of reconstructed regional beta-band oscillatory activity on one spatial direction (red trace) and its amplitude envelope estimated with the Hilbert transform (blue trace). A temporal segment with duration of 5 s is shown. **Lower panels**: Estimation of the DFA scaling exponents for regional amplitude envelope signals is exemplified using data from one participant in the study. The DFA scaling exponent represents the slope of the fluctuation function denoted by *F(time)* and was estimated using linear regression on a log-log graph with 10 equally spaced points between 1 s and 30 s. Fitting is illustrated for three cortical regions of each hemisphere that are shown with corresponding colors on the cortical surface, i.e., *superior parietal*, *caudal middle frontal* and *rostral middle frontal*. Across these regions, scaling exponents decrease from posterior to anterior regions in each hemisphere.

The time course of cortical activation was estimated for each of the *N* = 134 cortical regions of a modified Desikan–Killiany anatomical atlas [43] using a three-step approach, as explained in [21]. First, we applied a component-wise dimensionality reduction in each cortical region by extracting the first principal component of the reconstructed activity for each of the three spatial directions. Second, we determined the amplitude envelopes (AEs) of the three principal components as the absolute value of the analytic signal computed with the Hilbert transform (exemplified in the upper panel of Figure 1). Third, one representative AE time-series was subsequently estimated for each region as the root mean square of the three directional AEs at each time sample.

### 2.3. Detrended Fluctuation Analysis

The scale-free property of a signal refers to the absence of a characteristic temporal scale (or oscillation frequency) dominating the dynamics of the underlying process. Scale-free signals with a power-law behavior can be described by the exponent of a power-law function, which quantifies the power of the signal fluctuations as a function of the temporal scale at which those fluctuations are evaluated. In general, a scale-free behavior reflects a tendency of complex systems to develop long-range temporal correlations or dependencies, as measured by the decay of the temporal autocorrelation function. Previous studies have shown that amplitude envelopes of the brain oscillatory activity manifest scale-free, power-law behavior in the low-frequency range [15,44,45,46,47]. In our study, the scale-free behavior of the AEs of beta-band activity was characterized using the detrended fluctuation analysis (DFA, [44]). DFA estimates the scaling of the root mean square fluctuation of the integrated and linearly detrended AEs as a function of time-window size. The DFA *power-law* or *scaling* exponents, which represent the slope of the mean square fluctuation function, were calculated using linear regression on a log-log graph with 10 equally spaced points corresponding to temporal windows between 1 s and 30 s (Figure 1). The fit interval was similar with intervals used in previous studies [46,47]. We used a 50% overlap between consecutive windows for each size of the temporal window. The goodness-of-fit was calculated using the R-squared statistic. In general, a temporally uncorrelated signal is characterized by a DFA scaling exponent of 0.5, whereas larger scaling exponents in the interval of 0.5 to 1.0 that are typical for AE signals indicate the presence of LRTC [44]. A process that is correlated over longer time scales shows a tendency to both stay elevated for relatively longer time intervals and to remain at low levels for longer intervals. 

### 2.4. Relationship Between Local Scale-Free Dynamics and Inter-Hemispheric Coupling 

Our findings (see Section 3) indicated that the spatial distribution of the scaling coefficients resembles the previously reported regional variation in the correlation of spontaneous low-frequency hemodynamic fluctuations between homotopic areas (i.e., homologous anatomical regions) of the two hemispheres in healthy individuals during rest [48]. This similarity may reflect the fact that stronger coupling between homologous areas of the two hemispheres is associated with higher values of the DFA scaling exponents that characterize the local temporal dynamics of activity in those areas. This hypothesis is supported by computational models showing that (1) coupling between neuronal networks increases the similarity of the regional DFA exponents, and (2) dynamical states characterized by higher scaling exponents of the scale-free fluctuations optimize the coupling of the amplitude envelopes in neuronal networks [19]. 

Based on these observations, we conducted two analyses focusing on (1) the relationship between the regional scaling coefficients and the corresponding inter-hemispheric coupling between the low-frequency components of the AE from homologous cortical areas of the left and right hemispheres (referred hence to forth as inter-hemispheric coupling, IHC), and (2) the relationship between PTSS and IHC. For these analyses, we filtered the AEs in a frequency range between 0.033 Hz and 1 Hz that corresponds to the temporal window durations used in our study for the estimation of the power-law scaling coefficients. The IHC was characterized using Pearson correlations between the filtered AEs from homologous cortical areas of the left and right hemispheres. 

### 2.5. Statistical Analysis

Our previous study on functional connectivity [21] demonstrated that PTSS is associated with attenuated fluctuations in the very low frequency range, which is consistent with a possible reduction in long-range temporal correlations in the beta frequency band that would be reflected in lower scaling exponents. To test this hypothesis, we conducted a statistical analysis to ascertain if PTSS is associated with lower DFA scaling exponents. For this purpose, we used one-tailed nonparametric Kendall tests to investigate if there is a significant negative correlation between PCL5 scores and scaling exponents in each brain region. We opted to use PTSD symptom severity (as characterized by PCL-5 scores) as a continuous measure to maintain granular variance in symptom severity and avoid arbitrary diagnostic cutoffs associated with binary diagnostic groups. Results from these statistical tests were considered significant if the number of brain regions with uncorrected *p* < 0.05 was higher than the one that can be obtained by chance. Omnibus tests are particularly powerful when there are widespread but subtle effects across many tests with *p* < 0.05, being adopted by earlier electrophysiological studies focusing on the scale-free dynamics of brain activity (e.g., [46,47]). We used an omnibus test based on the empirical null distribution of the number of regions with *p* < 0.05 obtained using participant-level permutation analysis (with 10,000 permutations) that preserved the covariance structure across regions. The total number of regions with *p* < 0.05 from the original data was considered significant if it was greater than the 95th percentile of the null distribution. A supplementary analysis was also carried out to identify and report the regions with adjusted *p*-values lower than 0.05 after correction to control the false discovery rate (FDR) at q = 0.05.

A second statistical analysis was conducted to test the hypothesis that the regional scaling coefficients are related to IHC. For this purpose, the scaling coefficients were first averaged for each pair of homotopic areas. Subsequently, we used one-tailed Kendall correlation tests to investigate if there is a significant positive correlation between the averaged scaling coefficients and IHC for each pair of cortical regions, using observations of previous computational studies [19] that have demonstrated a positive correlation between scaling exponents and functional coupling. In a third statistical analysis, we used one-tailed Kendall correlation tests to investigate if there is a negative association between PTSS and IHC for each pair of regions. Results from each of these statistical analyses were considered significant if the number of homotopic regions with uncorrected *p* < 0.05 was higher than chance based on the empirical null distribution of the number of regions with *p* < 0.05 determined, as explained before.

## 3. Results

### 3.1. DFA Results

The amplitude modulation of the regional beta-band activity was well characterized by the power-law model over the range of temporal window sizes used in our analysis (Figure 1), consistent with findings reported by previous studies. Across cortical regions, the linear-fit model in log-log coordinates accounted, on average, for 99.7% to 99.8% of the data variance (the minimum R-squared across participants and regions was 97.8%). 

**Figure 2:**
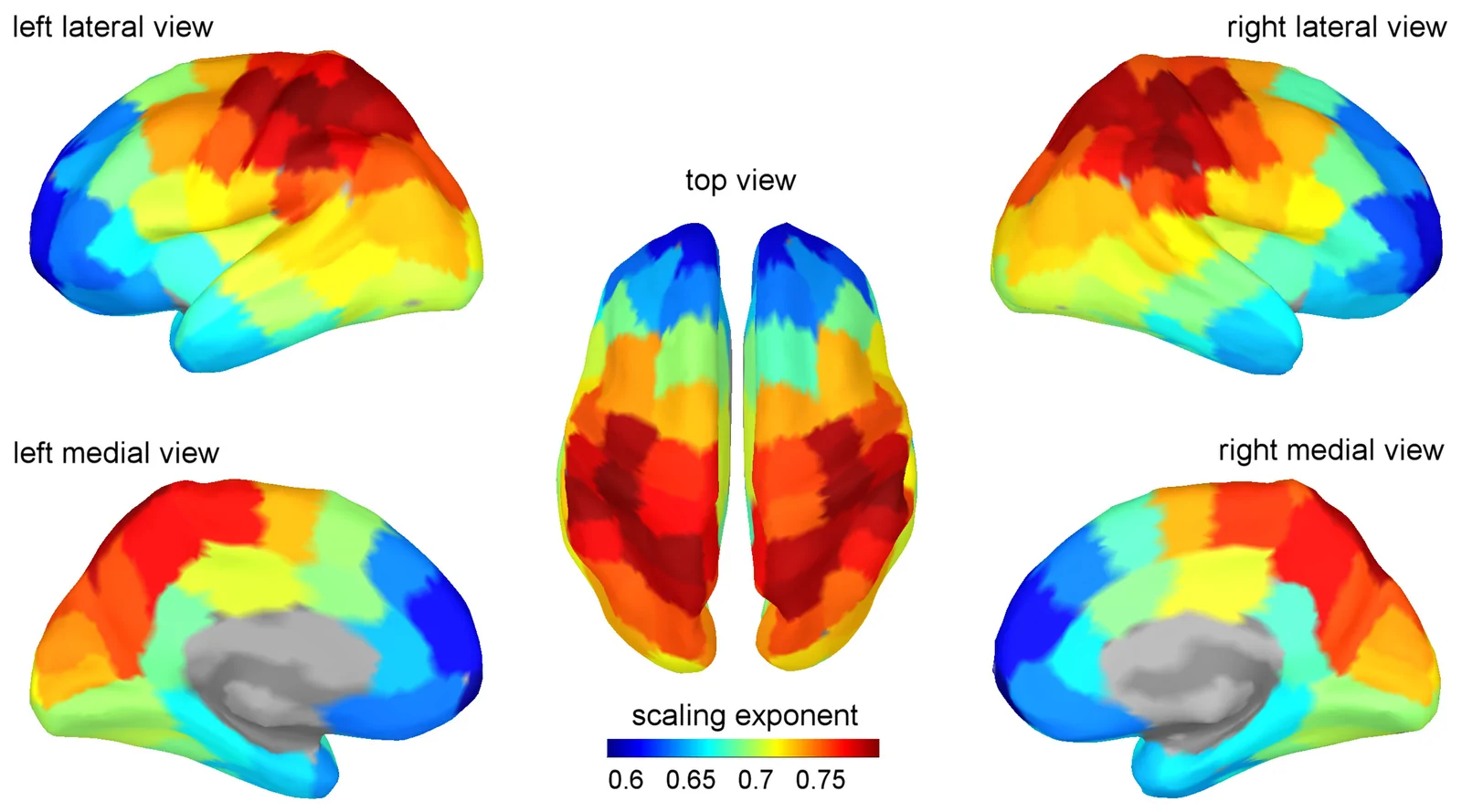
Regional mean DFA scaling exponents across all participants. The largest mean DFA scaling exponents were observed bilaterally over somatomotor and superior parietal regions, whereas the smallest mean DFA scaling exponents are seen bilaterally over frontal regions.

The spatial distribution of the mean scaling exponents across participants is shown in Figure 2. This spatial distribution is characterized by an inter-hemispheric symmetry with the largest mean scaling coefficients being observed bilaterally over somatomotor and superior parietal regions, and the smallest mean scaling exponents seen bilaterally over frontal regions. The inter-regional differences found in our study are in general agreement with previous observations of an EEG study on healthy individuals that reported the largest scaling exponents determined from fluctuations in the AE of beta-band oscillations at central and parietal electrodes, and the smallest scaling exponents at frontal electrodes [45].

### 3.2. Correlation Between PTSS and Scaling Exponents

**Figure 3:**
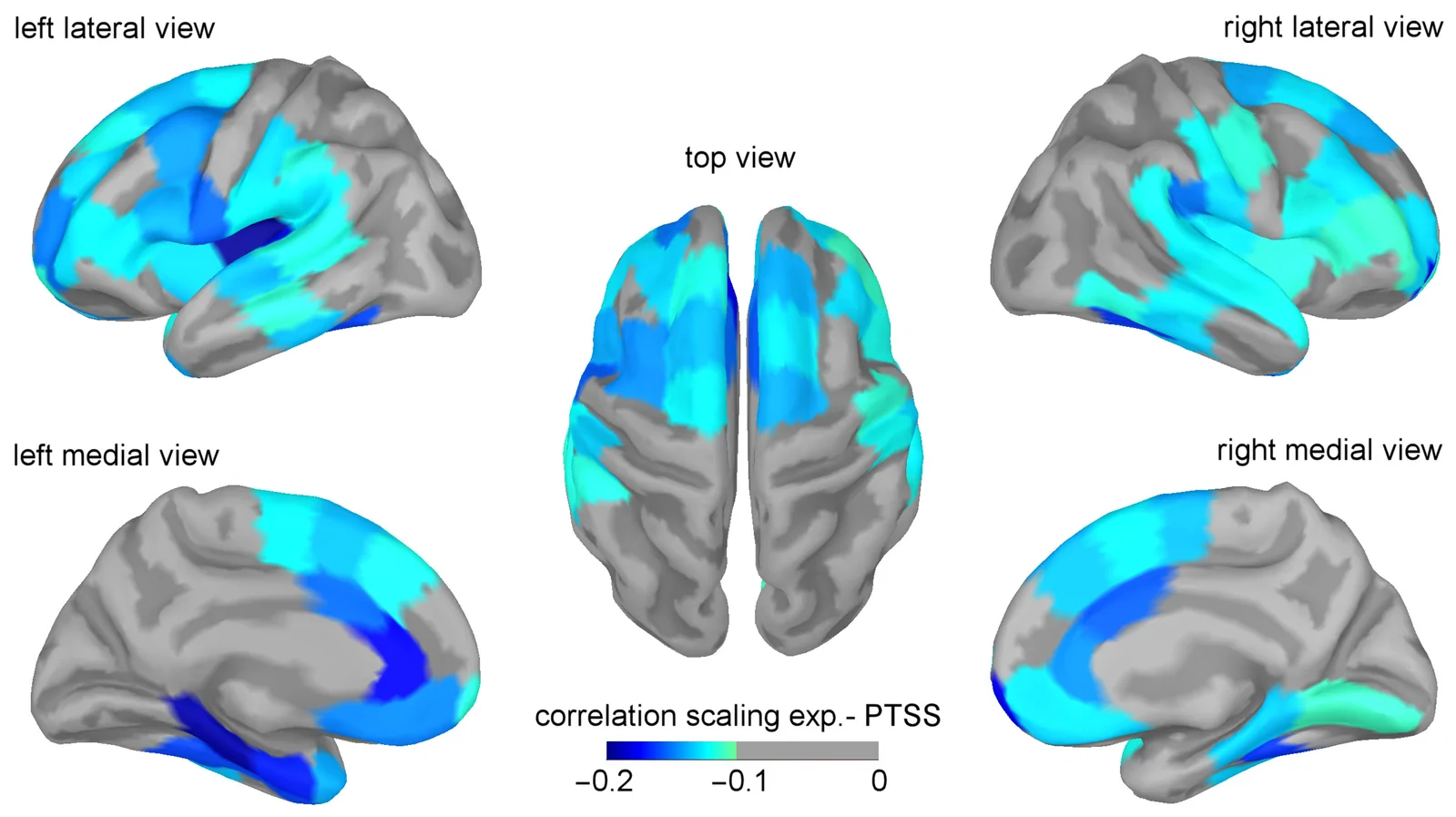
Correlations between PTSS and DFA scaling exponents. Significant negative correlations with uncorrected *p* < 0.05 were present for 61 cortical regions that are shown in color in different views of the brain. The strongest correlations are seen for the left posterior insula, the left parahippocampal cortex, and the left rostral anterior cingulate cortex.

Significant negative correlations between PTSS and scaling exponents with uncorrected *p* < 0.05 were present in *n* = 61 cortical regions shown in Figure 3, which represented a higher-than-chance number of brain regions (*p* = 0.029). The supplementary analysis indicated that the observed effects were widespread but rather subtle as none of these regions showed strong effects that would be significant after correction to control the FDR. These regions were located predominantly in the ventromedial and dorsolateral prefrontal cortex, insula, and association cortex of the temporal lobe. The strongest correlations (*p* < 0.005) were present for the left posterior insula (*r* = −0.204, *p* = 0.0018), the left parahippocampal cortex (*r* = −0.196, *p* = 0.0025), and the left rostral anterior cingulate cortex (*r* = −0.181, *p* = 0.005). Among all regions that showed significant correlations between PTSS and scaling exponents, none showed significant correlations between PTSS and the goodness-of-fit (all *p* > 0.05).

### 3.3. Correlation Between Local Scale-Free Dynamics and Inter-Hemispheric Coupling

As we observed earlier, the regional distribution of the mean scaling exponents in each hemisphere shows relatively large values in sensory-motor cortices, comparatively smaller values in primary and unimodal association areas, and the smallest values in high-order heteromodal processing areas of the temporal and frontal lobes (Figure 2). This spatial distribution of the scaling exponents resembles the regional variation in the correlation of resting-state hemodynamic fluctuations between homotopic areas of the two hemispheres in healthy individuals during rest [48]. 

**Figure 4:**
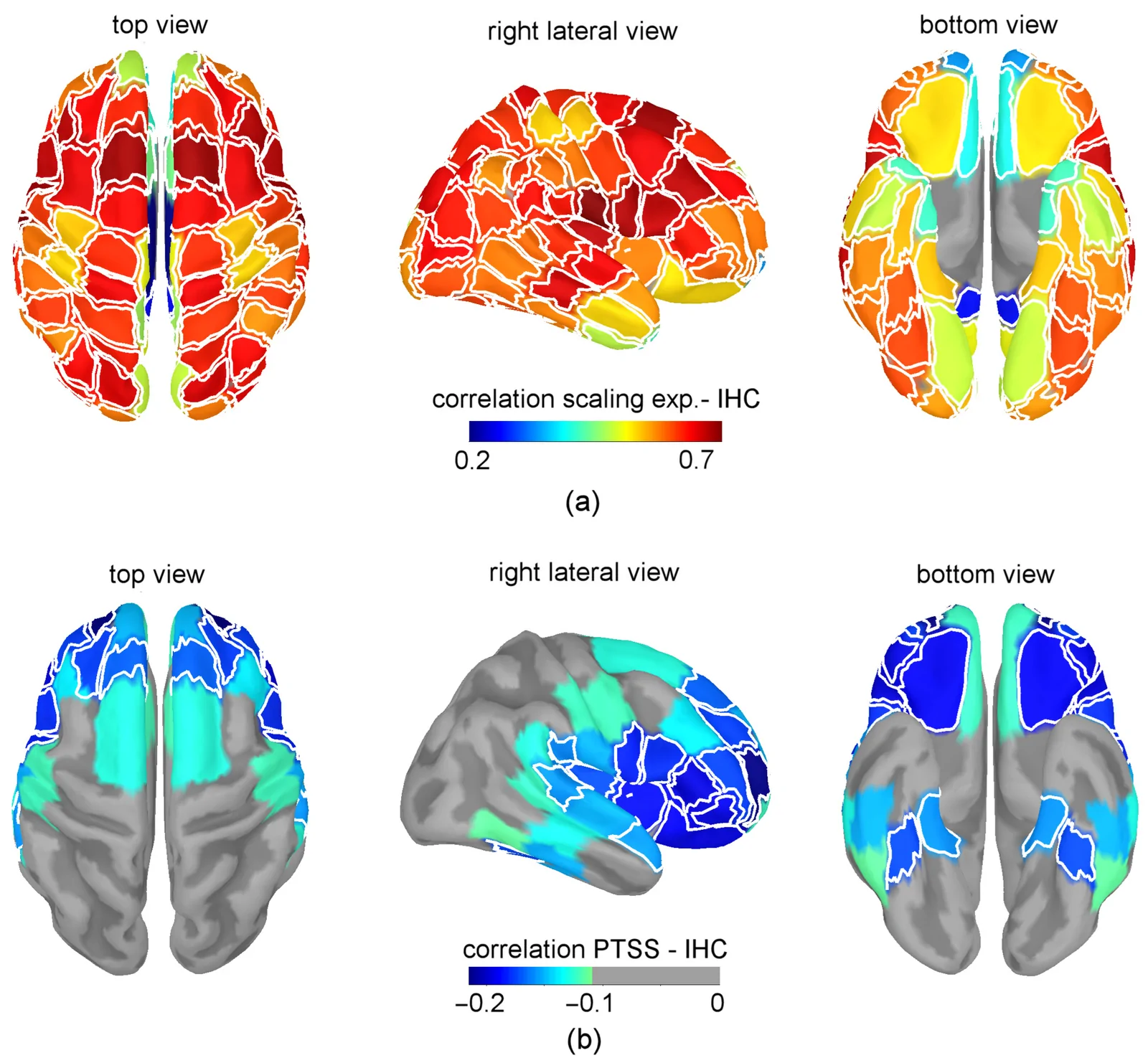
(**a**) Cortical areas with significant correlations at uncorrected *p* < 0.05 between the DFA scaling exponents and inter-hemispheric coupling (IHC) are shown in different views of the brain. White borders are used to mark the regions with significant correlations after correction to control the FDR. (**b**) Cortical areas with significant correlations at uncorrected *p* < 0.05 between post-traumatic stress severity (PTSS) and IHC. IHC was negatively correlated with PTSS (*p* < 0.05) for 33 pairs of homotopic cortical areas predominantly from prefrontal and temporal lobes. White borders are used to mark the regions with significant correlations after correction to control the FDR.

The statistical analysis conducted to investigate the relationship between the regional scaling exponents and IHC showed that all pairs of regions had significant positive correlations at uncorrected *p* < 0.05 (Figure 4a), with all but one region (posterior cingulate) showing also significant results after correction to control the FDR. Regions with lower correlations between scaling exponents and IHC included several areas of the anterior and medial prefrontal cortex, anterior lateral and medial temporal lobe, medial occipital cortex, and anterior insula. Overall, these results indicate stronger association between scaling exponents and IHC across primary and unimodal association cortices and weaker association across some of the high-order heteromodal processing areas.

### 3.4. Correlation Between PTSS and Inter-Hemispheric Coupling

The statistical analysis conducted to investigate the relationship between PTSS and IHC showed that 33 pairs of regions (which represent 49% of the total number of 67 pairs) had negative correlations between PTSS and IHC with uncorrected *p* < 0.05, which represented a higher-than-chance number of brain regions (*p* = 0.018). A subset of 16 regions (shown with borders in Figure 4b) showed strong effects that were also significant after correction to control the FDR. These regions were located predominantly in prefrontal and temporal lobes and included areas of the superior, middle and inferior frontal gyri, lateral orbitofrontal cortex, insula, fusiform and parahippocampal cortex, lateral temporal cortex, supramarginal gyrus and motor cortex.

## 4. Discussion

We found that higher PTSS is associated with lower scaling exponents determined from regional AEs of beta-band activity, suggesting a deviation from an optimal state of neuronal network criticality. While scale-free dynamics and long-range temporal correlations are hallmark features of critical brain networks, it is important to note that the DFA exponent represents only one proxy for these complex dynamics. Therefore, the observed reduction in DFA exponents should not be equated directly with definitive evidence of a breakdown in neuronal criticality. Instead, our results are more accurately interpreted as an alteration in the system’s temporal scaling properties, reflecting a deviation from the optimal, scale-invariant regime typically observed in healthy states. The lower scaling exponents with PTSS were not associated with a departure from the linear model, which shows that for patients with high PTSS the temporal dynamics of the infraslow AE fluctuations is still well characterized by scale-free behavior, but with a reduced ability to optimally generate or dissipate synchronized activity (coupling) in neural networks over long temporal scales. This finding extends the observations from a previous EEG study that also reported lower scaling exponents for the AEs of alpha-band oscillations in PTSD [47]. Our study also provides evidence that alterations in local scale-free dynamics are associated with lower coupling of the infraslow AE fluctuations between homotopic areas of the two hemispheres. Lower DFA exponents and inter-hemispheric connectivity were observed in numerous regions that include the insula, anterior cingulate cortex, parahippocampal cortex, and prefrontal areas. These regions anchor key fear-processing and salience-processing circuits, and their dysfunction can affect contextual processing and top-down emotional regulation in PTSD [49,50]. Furthermore, they belong to core functional networks, such as the salience, default mode, and central executive networks, whose breakdown has been linked to PTSD in a number of influential neurobiological models [51]. 

Computational studies have shown that scale-free local activity emerges in neuronal networks for a specific range of excitatory to inhibitory connections [18,52]. Consistent with this finding, changes in scale-free activity associated with neurological disorders can arise from imbalances between neuronal excitation and inhibition. DFA scaling exponents quantify the long-range temporal correlations so that large exponents indicate stronger dependencies over time. DFA scaling exponents decrease both toward inhibition-dominated (subcritical) and excitation-dominated (supercritical) states [15,18]. In one of our recent studies, we found that PTSD is also associated with alterations in arrhythmic cortical activity that are consistent with a relatively lower level of inhibitory activity [53]. One possible explanation for a chronic disruption in excitatory to inhibitory balance would be alterations in one or more neurotransmitter system functions, induced by acute or chronic stress ([54] for a review), which would also contribute to the observed changes in the power-law behavior of the amplitude of beta-band oscillations in PTSD.

Previous studies have demonstrated that states characterized by scale-free activity and long-range temporal correlations maximize the normal brain’s dynamic range and information-processing capabilities [55]. There have been reports of an abnormal dynamic range in sensory processing in PTSD. Specifically, patients with PTSD do not show the typical increase in the amplitude of the sensory evoked-response components as stimulus intensity increases [56,57]. Experimental and computational studies have shown that the amplitude of the evoked responses is modulated by spontaneous brain activity and exhibits the largest dynamic range when ongoing brain activity is characterized by critical dynamics [55,58,59]. Hence, our findings may provide support for the premise that the abnormal stimulus intensity–response amplitude functions in PTSD can be due at least in part to alterations in the dynamics of spontaneous brain activity. Future studies could directly test the potential relationship between the deviation from an optimal state of neuronal network criticality and reduced sensory dynamic range in PTSD. A decreased sensory dynamic range reflecting the inability of a neuronal network to map different inputs onto distinguishable network outputs could contribute to other sensory processing deficits that are typically observed in individuals with PTSD [60]. Notably, it could contribute to a core symptom of PTSD, the high frequency of intrusive memories of the traumatic event, through deficits in the function of pattern separation and the phenomenon of overgeneralization of trauma reminders. In the setting of these pathological processes, sensory stimuli that share even vague visual features with stimuli that were perceived in conjunction with the traumatic event can trigger the reactivation of traumatic memories, indicative of a dysfunctional sensory input to memory output transformation, or abnormal pattern completion.

Our findings also suggest that alterations in scale-free dynamics are linked to lower inter-hemispheric coupling of the infraslow AE fluctuations with PTSS. Inter-hemispheric connectivity is critically important for sensory and motor coordination and the integration of complex cognitive processes associated with executive function, long-term memory, and spatial attention. Therefore, disruptions of IHC are associated with neurodevelopmental and neuropsychiatric disorders [61]. Lower inter-hemispheric functional connectivity associated with brain disorders has been traditionally viewed as a result of structural abnormalities of the long-range connections between the homotopic regions of the two hemispheres. The association between local scale-free dynamics and IHC that we found in our study aligns with results of computational studies that have reported a correlation between scaling exponents of the scale-free local activity and long-range functional coupling in neuronal networks [19]. Those studies demonstrated that networks functioning in a critical state are most responsive to inputs from distant brain regions and alterations in the scale-free dynamics of local brain activity can lead to impaired long-range coupling. We found a strong association between scaling exponents and IHC across primary and unimodal association cortices, i.e., within bilateral networks that are engaged in sensory integration and motor coordination, and weaker association across high-order heteromodal processing areas, potentially reflecting a higher degree of functional lateralization of these regions. Our finding of lower IHC with PTSS is consistent with a previous report of alterations in voxel-mirrored homotopic connectivity in resting-state fMRI data in PTSD [62]. Notably, the results reported by Sun et al. were obtained from participants who were evaluated soon (i.e., within two days) after the exposure to psychological trauma, suggesting that these alterations could represent a pre-disposing risk-factor (with a genetic or developmental origin) for the development of PTSD upon exposure to psychologically traumatic events. The correlation between DFA scaling coefficients and IHC is consistent with the fact that local alterations in the scale-free cortical activity contribute to dysfunction of large-scale functional connectivity in PTSD. However, this does not exclude the potential contribution of additional factors to IHC dysfunction, particularly for those individuals who also sustained brain injuries. Among these factors are abnormalities in the volume of the corpus callosum [63,64] or inter-hemispheric structural connectivity that were also reported in PTSD by studies using DTI [65,66].

Our results suggest that the reduced functional connectivity and altered LRTC could represent distinct but inter-connected hallmark features of a breakdown of the spatiotemporal brain dynamics in PTSD. These findings add to recent evidence from a growing number of resting-state MEG studies that suggest that clinically relevant abnormalities in spatiotemporal brain activity can be characterized by the persistence of local amplitude fluctuations quantified by DFA exponents, connectome fingerprinting that evaluates individual identification profiles and within-participant spatial stability of large-scale functional networks over time, and neuronal avalanches that characterize the propagation of transient activity across large-scale brain networks. For example, several MEG studies have demonstrated that Alzheimer’s disease progression is coupled with a progressive shift toward supercritical brain dynamics characterized by changes in LRTC [46,67,68]. Similarly, attenuations of LRTCs have been reported in MEG studies in major depressive disorder [69] and schizophrenia [70,71]. MEG studies in multiple sclerosis (MS) have demonstrated that reduced individual functional connectome identifiability and stability are linked to fatigue severity [72], and neuronal-avalanche repertoires relate to neurological impairment and differ across MS phenotypes [73]. A study in mild cognitive impairment has also shown that changes in the topology of large-scale neuronal-avalanche dynamics may predict early memory dysfunction [74]. Avalanche-transition analyses have also identified altered propagation of fast aperiodic activity associated with disease duration in amyotrophic lateral sclerosis [75]. The findings of these studies indicate that DFA scaling exponents, connectome fingerprinting, and neuronal-avalanche analysis may provide clinically actionable biomarkers of the breakdown of the spatiotemporal brain dynamics in neurodegenerative and psychiatric disorders.

Some limitations of our study arise from the fact that all participants were adult males with combat exposure who had a history of mTBI, and some of them were taking medications with central nervous system effects. Future studies are necessary to understand if our results generalize to other populations and types of psychological trauma. While our analyses focused exclusively on the IHC between homotopic regions, similar associations might also be present for other large-scale functional networks that were not the subject of this investigation. Furthermore, our study demonstrates the association between PTSS, scaling exponents and IHC, but could not determine the causality between these alterations and the development of PTSD symptoms due to its cross-sectional design. Some of our findings may also reflect the combined effects of PTSD and mTBI. In the absence of a priori knowledge of causality relationships between PTSS and measures of brain activity, our study has focused exclusively on the strength of the association between these variables, without implying causation. Future longitudinal studies that could provide greater insights into potential causal relationships may enable the use of more complex predictive models to determine whether the observed associations remain significant after adjusting for other factors with possible influence on the outcome variables or to explore the statistical mediation between alterations in scale-free dynamics, functional connectivity and PTSD symptoms. It is also important to note that while the observed correlations between PTSS and scaling exponents in this study reached statistical significance, the effect sizes were relatively modest. Neuroimaging studies frequently observe effect sizes of this magnitude, suggesting that other multifaceted factors are also at play. Nevertheless, the correlations observed may still be relevant as they may provide valuable insights into previously unexplored mechanistic pathways that can be exploited for the benefit of patients with neurological and psychiatric disorders. A limitation of the current study is also the lack of a test–retest dataset to evaluate the reliability and reproducibility of our extracted MEG metrics over time. While formal reliability metrics are unavailable for our sample of participants, prior studies in healthy participants have demonstrated that long-range temporal correlations exhibit significant reliability across sessions separated by several days [76]. 

## 5. Conclusions

To conclude, our findings are consistent with the hypothesis that reduced coupling of the beta-band AEs in PTSD may be due in part to alterations in the mechanisms that govern the local scale-free dynamics of the regional infraslow AE fluctuations. A plausible interpretation of the reduced DFA scaling exponents in PTSD is that they reflect alterations in excitation–inhibition balance, which is consistent with previous computational and experimental work. According to this interpretation, our observations suggest that pharmacological or non-pharmacological therapies that can re-establish the balance between excitation and inhibition and/or enhance the very low frequency fluctuations of the beta-band amplitude envelopes may restore the scale-free dynamics of the local brain activity. For example, neurofeedback using closed-loop brain training has been shown to increase the DFA exponents after training [47], and neuromodulation using rTMS, which can influence the excitability of a targeted cortical region depending on the stimulation frequency [77], has demonstrated beneficial effects on PTSD symptoms [78,79,80,81]. Future studies can investigate if these therapies may be beneficial for treating or preventing PTSD partly because of their potential to restore the long-range functional coupling of neuronal activity, including the inter-hemispheric connectivity that is critically important for brain function. Future research using electrophysiological recordings before and after treatment is necessary to determine if the scale-free dynamics of local cortical activity can be used to identify patients who are more or less likely to benefit from specific therapies for PTSD and to track a patient’s response to therapy.

## 6. Disclaimers

The views expressed in this article are those of the authors and do not necessarily reflect the official policy of the Defense Health Agency, Department of War or the U.S. Government. The identification of specific products or scientific instrumentation is considered an integral part of the scientific endeavor and does not constitute endorsement or implied endorsement on the part of the authors, DoW, or any component agency. The study protocol was approved by the Walter Reed National Military Medical Center in compliance with all applicable Federal regulations governing the protection of human subjects. This material has been reviewed by the Walter Reed Army Institute of Research. There is no objection to its publication. The investigators have adhered to policies for protection of human subjects as prescribed in AR 70-25.

## Data Availability

The data supporting the findings of this article can be made available on request, contingent on institutional approval.

## References

[1] Goldstein R.B., Smith S.M., Chou S.P., Saha T.D., Jung J., Zhang H., Pickering R.P., Ruan W.J., Huang B., Grant B.F. (2016). The epidemiology of DSM-5 posttraumatic stress disorder in the United States: Results from the National Epidemiologic Survey on Alcohol and Related Conditions-III. Soc. Psychiatry Psychiatr. Epidemiol..

[2] Kessler R.C., Sonnega A., Bromet E., Hughes M., Nelson C.B. (1995). Posttraumatic stress disorder in the national comorbidity survey. Arch. Gen. Psychiatry.

[3] Koenen K.C., Ratanatharathorn A., Ng L., McLaughlin K.A., Bromet E.J., Stein D.J., Karam E.G., Ruscio A.M., Benjet C., Scott K. (2017). Posttraumatic stress disorder in the World Mental Health Surveys. Psychol. Med..

[4] Dohrenwend B.P., Turner J.B., Turse N.A., Adams B.G., Koenen K.C., Marshall R. (2006). The psychological risks of Vietnam for U.S. veterans: A revisit with new data and methods. Science.

[5] Schell T.L., Marshall G.N., Tanielian T., Jaycox L.H. (2008). Survey of individuals previously deployed for OEF/OIF. Invisible Wounds of War: Psychological and Cognitive Injuries, Their Consequences, and Services to Assist Recovery.

[6] Schein J., Houle C., Urganus A., Cloutier M., Patterson-Lomba O., Wang Y., King S., Levinson W., Guérin A., Lefebvre P. (2021). Prevalence of post-traumatic stress disorder in the United States: A systematic literature review. Curr. Med. Res. Opin..

[7] Bryant R.A., Creamer M., O’donnell M., Silove D., Clark R., Mcfarlane A.C. (2009). Post-traumatic amnesia and the nature of post-traumatic stress disorder after mild traumatic brain injury. J. Int. Neuropsychol. Soc..

[8] Bryant R.A., O’Donnell M.L., Creamer M., McFarlane A.C., Clark C.R., Silove D. (2010). The psychiatric sequelae of traumatic injury. Am. J. Psychiatry.

[9] Hoge C.W., McGurk D., Thomas J.L., Cox A.L., Engel C.C., Castro C.A. (2008). Mild traumatic brain injury in U.S. Soldiers returning from Iraq. N. Engl. J. Med..

[10] Schneiderman A.I., Braver E.R., Kang H.K. (2008). Understanding sequelae of injury mechanisms and mild traumatic brain injury incurred during the conflicts in Iraq and Afghanistan: Persistent postconcussive symptoms and posttraumatic stress disorder. Am. J. Epidemiol..

[11] Stein M.B., Kessler R.C., Heeringa S.G., Jain S., Campbell-Sills L., Colpe L.J., Fullerton C.S., Nock M.K., Sampson N.A., Schoenbaum M. (2015). Prospective Longitudinal Evaluation of the Effect of Deployment-Acquired Traumatic Brain Injury on Posttraumatic Stress and Related Disorders: Results From the Army Study to Assess Risk and Resilience in Service members (Army STARRS). Am. J. Psychiatry.

[12] Yurgil K.A., Barkauskas D.A., Vasterling J.J., Nievergelt C.M., Larson G.E., Schork N.J., Litz B.T., Nash W.P., Baker D.G. (2014). Association between traumatic brain injury and risk of posttraumatic stress disorder in active-duty marines. JAMA Psychiatry.

[13] Beggs J.M., Plenz D. (2003). Neuronal avalanches in neocortical circuits. J. Neurosci..

[14] Haldeman C., Beggs J.M. (2005). Critical branching captures activity in living neural networks and maximizes the number of metastable States. Phys. Rev. Lett..

[15] Linkenkaer-Hansen K., Nikouline V.V., Palva J.M., Ilmoniemi R.J. (2001). Long-range temporal correlations and scaling behavior in human brain oscillations. J. Neurosci..

[16] Petermann T., Thiagarajan T.C., Lebedev M.A., Nicolelis M.A., Chialvo D.R., Plenz D. (2009). Spontaneous cortical activity in awake monkeys composed of neuronal avalanches. Proc. Natl. Acad. Sci. USA.

[17] Smit D.J., de Geus E.J., van de Nieuwenhuijzen M.E., van Beijsterveldt C.E., van Baal G.C., Mansvelder H.D., Boomsma D.I., Linkenkaer-Hansen K. (2011). Scale-free modulation of resting-state neuronal oscillations reflects prolonged brain maturation in humans. J. Neurosci..

[18] Poil S.S., Hardstone R., Mansvelder H.D., Linkenkaer-Hansen K. (2012). Critical-state dynamics of avalanches and oscillations jointly emerge from balanced excitation/inhibition in neuronal networks. J. Neurosci..

[19] Avramiea A.E., Masood A., Mansvelder H.D., Linkenkaer-Hansen K. (2022). Long-range amplitude coupling is optimized for brain networks that function at criticality. J. Neurosci..

[20] Koch S.B., van Zuiden M., Nawijn L., Frijling J.L., Veltman D.J., Olff M. (2016). Aberrant Resting-State Brain Activity In Posttraumatic Stress Disorder: A Meta-Analysis And Systematic Review. Depress. Anxiety.

[21] Popescu M., Popescu E.A., DeGraba T.J., Hughes J.D. (2024). Altered long-range functional connectivity in PTSD: Role of the infraslow oscillations of cortical activity amplitude envelopes. Clin. Neurophysiol..

[22] Ross M.C., Cisler J.M. (2020). Altered large-scale functional brain organization in posttraumatic stress disorder: A comprehensive review of univariate and network-level neurocircuitry models of PTSD. NeuroImage Clin..

[23] Shim M., Im C.H., Lee S.H. (2017). Disrupted cortical brain network in posttraumatic stress disorder patients: A resting-state electroencephalographic study. Transl. Psychiatry.

[24] Toll R.T., Wu W., Naparstek S., Zhang Y., Narayan M., Patenaude B., De Los Angeles C., Sarhadi K., Anicetti N., Longwell P. (2020). An Electroencephalography Connectomic Profile of Posttraumatic Stress Disorder. Am. J. Psychiatry.

[25] Betti V., Della Penna S., de Pasquale F., Corbetta M. (2021). Spontaneous Beta Band Rhythms in the Predictive Coding of Natural Stimuli. Neuroscientist.

[26] Donner T.H., Siegel M. (2011). A framework for local cortical oscillation patterns. Trends Cogn. Sci..

[27] Spitzer B., Haegens S. (2017). Beyond the status quo: A role for beta oscillations in endogenous content (re)activation. eNeuro.

[28] Engel A.K., Fries P. (2010). Beta-band oscillations–Signalling the status quo?. Curr. Opin. Neurobiol..

[29] Blevins C.A., Weathers F.W., Davis M.T., Witte T.K., Domino J.L. (2015). The Posttraumatic Stress Disorder Checklist for DSM-5 (PCL-5): Development and Initial Psychometric Evaluation. J. Trauma. Stress.

[30] Bovin M.J., Marx B.P., Weathers F.W., Gallagher M.W., Rodriguez P., Schnurr P.P., Keane T.M. (2015). Psychometric properties of the PTSD Checklist for Diagnostic and Statistical Manual of Mental Disorders-Fifth Edition (PCL-5) in Veterans. Psychol. Assess..

[31] American Congress of Rehabilitation Medicine (1993). Definition of mild traumatic brain injury. J. Head Trauma Rehabil..

[32] Bush K., Kivlahan D.R., McDonell M.B., Fihn S.D., Bradley K.A. (1998). The AUDIT alcohol consumption questions (AUDIT-C): An effective brief screening test for problem drinking. Ambulatory Care Quality Improvement Project (ACQUIP). Alcohol Use Disorders Identification Test. Arch. Intern. Med..

[33] Kroenke K., Spitzer R.L., Williams J.B. (2001). The PHQ-9: Validity of a brief depression severity measure. J. Gen. Intern. Med..

[34] Rytwinski N.K., Scur M.D., Feeny N.C., Youngstrom E.A. (2013). The co-occurrence of major depressive disorder among individuals with posttraumatic stress disorder: A meta-analysis. J. Trauma. Stress.

[35] Flory J.D., Yehuda R. (2015). Comorbidity between post-traumatic stress disorder and major depressive disorder: Alternative explanations and treatment considerations. Dialogues Clin. Neurosci..

[36] Delorme A., Makeig S. (2004). EEGLAB: An open source toolbox for analysis of single-755 trial EEG dynamics including independent component analysis. J. Neurosci. Methods.

[37] Gross J., Baillet S., Barnes G.R., Henson R.N., Hillebrand A., Jensen O., Jerbi K., Litvak V., Maess B., Oostenveld R. (2013). Good practice for conducting and reporting MEG research. NeuroImage.

[38] Fischl B. (2012). FreeSurfer. NeuroImage.

[39] Tadel F., Baillet S., Mosher J.C., Pantazis D., Leahy R.M. (2011). Brainstorm: A User-Friendly Application for MEG/EEG Analysis. Comput. Intell. Neurosci..

[40] Hämäläinen M.S., Ilmoniemi R.J. (1994). Interpreting magnetic fields of the brain: Minimum norm estimates. Med. Biol. Eng. Comput..

[41] Huang M.X., Mosher J.C., Leahy R.M. (1999). A sensor-weighted overlapping-sphere head model and exhaustive head model comparison for MEG. Phys. Med. Biol..

[42] Leahy R.M., Mosher J.C., Spencer M.E., Huang M.X., Lewine J.D. (1998). A study of dipole localization accuracy for MEG and EEG using a human skull phantom. Electroencephalogr. Clin. Neurophysiol..

[43] Desikan R.S., Ségonne F., Fischl B., Quinn B.T., Dickerson B.C., Blacker D., Buckner R.L., Dale A.M., Maguire R.P., Hyman B.T. (2006). An automated labeling system for subdividing the human cerebral cortex on MRI scans into gyral based regions of interest. NeuroImage.

[44] Hardstone R., Poil S.-S., Schiavone G., Jansen R., Nikulin V.V., Mansvelder H.D., Linkenkaer-Hansen K. (2012). Detrended fluctuation analysis: A scale-free view on neuronal oscillations. Front. Physiol..

[45] Linkenkaer-Hansen K., Smit D.J., Barkil A., van Beijsterveldt T.E., Brussaard A.B., Boomsma D.I., van Ooyen A., de Geus E.J. (2007). Genetic contributions to long-range temporal correlations in ongoing oscillations. J. Neurosci..

[46] Montez T., Poil S.S., Jones B.F., Manshanden I., Verbunt J.P., van Dijk B.W., Brussaard A.B., van Ooyen A., Stam C.J., Scheltens P. (2009). Altered temporal correlations in parietal alpha and prefrontal theta oscillations in early-stage Alzheimer disease. Proc. Natl. Acad. Sci. USA.

[47] Ros T., Frewen P., Théberge J., Michela A., Kluetsch R., Mueller A., Candrian G., Jetly R., Vuilleumier P., Lanius R.A. (2017). Neurofeedback Tunes Scale-Free Dynamics in Spontaneous Brain Activity. Cereb. Cortex.

[48] Stark D.E., Margulies D.S., Shehzad Z.E., Reiss P., Kelly A.M., Uddin L.Q., Gee D.G., Roy A.K., Banich M.T., Castellanos F.X. (2008). Regional variation in interhemispheric coordination of intrinsic hemodynamic fluctuations. J. Neurosci..

[49] Fenster R.J., Lebois L.A.M., Ressler K.J., Suh J. (2018). Brain circuit dysfunction in post-traumatic stress disorder: From mouse to man. Nat. Rev. Neurosci..

[50] Liberzon I., Abelson J.L. (2016). Context Processing and the Neurobiology of Post-Traumatic Stress Disorder. Neuron.

[51] Akiki T.J., Averill C.L., Abdallah C.G. (2017). A Network-Based Neurobiological Model of PTSD: Evidence from Structural and Functional Neuroimaging Studies. Curr. Psychiatry Rep..

[52] Bruining H., Hardstone R., Juarez-Martinez E.L., Sprengers J., Avramiea A.E., Simpraga S., Houtman S.J., Poil S.S., Dallares E., Palva S. (2020). Measurement of excitation-inhibition ratio in autism spectrum disorder using critical brain dynamics. Sci. Rep..

[53] Popescu M., Popescu E.A., DeGraba T.J., Hughes J.D. (2026). Resting-state scale-free arrhythmic cortical activity in post-traumatic stress disorder. Psychophysiology.

[54] Huang J., Xu F., Yang L., Tuolihong L., Wang X., Du Z., Zhang Y., Yin X., Li Y., Lu K. (2023). Involvement of the GABAergic system in PTSD and its therapeutic significance. Front. Mol. Neurosci..

[55] Avramiea A.E., Hardstone R., Lueckmann J.M., Bím J., Mansvelder H.D., Linkenkaer-Hansen K. (2020). Pre-stimulus phase and amplitude regulation of phase-locked responses are maximized in the critical state. eLife.

[56] Lewine J.D., Thoma R.J., Provencal S.L., Edgar C., Miller G.A., Canive J.M. (2002). Abnormal stimulus-response intensity functions in posttraumatic stress disorder: An electrophysiological investigation. Am. J. Psychiatry.

[57] Paige S.R., Reid G.M., Allen M.G., Newton J.O. (1990). Psychophysiological correlates of post-traumatic stress disorder in Vietnam veterans. Biol. Psychiatry.

[58] Gautam S.H., Hoang T.T., McClanahan K., Grady S.K., Shew W.L. (2015). Maximizing sensory dynamic range by tuning the cortical state to criticality. PLoS Comput. Biol..

[59] Shew W.L., Yang H., Petermann T., Roy R., Plenz D. (2009). Neuronal avalanches imply maximum dynamic range in cortical networks at criticality. J. Neurosci..

[60] Fleming L.L., Harnett N.G., Ressler K.J. (2024). Sensory alterations in post-traumatic stress disorder. Curr. Opin. Neurobiol..

[61] Jin X., Liang X., Gong G. (2020). Functional Integration Between the Two Brain Hemispheres: Evidence From the Homotopic Functional Connectivity Under Resting State. Front. Neurosci..

[62] Sun Y.W., Hu H., Wang Y., Ding W.N., Chen X., Wan J.Q., Zhou Y., Wang Z., Xu J.R. (2015). Inter-hemispheric functional and anatomical connectivity abnormalities in traffic accident-induced PTSD: A study combining fMRI and DTI. J. Affect. Disord..

[63] Jackowski A.P., Douglas-Palumberi H., Jackowski M., Win L., Schultz R.T., Staib L.W., Krystal J.H., Kaufman J. (2008). Corpus callosum in maltreated children with posttraumatic stress disorder: A diffusion tensor imaging study. Psychiatry Res. Neuroimaging.

[64] Saar-Ashkenazy R., Cohen J.E., Guez J., Gasho C., Shelef I., Friedman A., Shalev H. (2014). Reduced corpus-callosum volume in posttraumatic stress disorder highlights the importance of interhemispheric connectivity for associative memory. J. Trauma. Stress.

[65] Graziano R., Bruce S., Paul R. (2021). The Corpus Callosum and PTSD Severity. J. Interpers. Violence.

[66] Mohamed A.Z., Cumming P., Nasrallah F.A. (2021). Department of Defense Alzheimer’s Disease Neuroimaging Initiative. White Matter Alterations Are Associated with Cognitive Dysfunction Decades After Moderate-to-Severe Traumatic Brain Injury and/or Posttraumatic Stress Disorder. Biol. Psychiatry Cogn. Neurosci. Neuroimaging.

[67] Javed E., Suárez-Méndez I., Susi G., Román J.V., Palva J.M., Maestú F., Palva S. (2025). A Shift Toward Supercritical Brain Dynamics Predicts Alzheimer’s Disease Progression. J. Neurosci..

[68] van Nifterick A.M., Mulder D., Duineveld D.J., Diachenko M., Scheltens P., Stam C.J., van Kesteren R.E., Linkenkaer-Hansen K., Hillebrand A., Gouw A.A. (2023). Resting-state oscillations reveal disturbed excitation-inhibition ratio in Alzheimer’s disease patients. Sci. Rep..

[69] Linkenkaer-Hansen K., Monto S., Rytsälä H., Suominen K., Isometsä E., Kähkönen S. (2005). Breakdown of long-range temporal correlations in theta oscillations in patients with major depressive disorder. J. Neurosci..

[70] Alamian G., Pascarella A., Lajnef T., Knight L., Walters J., Singh K.D., Jerbi K. (2020). Patient, interrupted: MEG oscillation dynamics reveal temporal dysconnectivity in schizophrenia. NeuroImage Clin..

[71] Nikulin V.V., Jönsson E.G., Brismar T. (2012). Attenuation of long range temporal correlations in the amplitude dynamics of alpha and beta neuronal oscillations in patients with schizophrenia. NeuroImage.

[72] Cipriano L., Lopez E.T., Liparoti M., Minino R., Romano A., Polverino A., Ciaramella F., Ambrosanio M., Bonavita S., Jirsa V. (2023). Reduced clinical connectome fingerprinting in multiple sclerosis predicts fatigue severity. NeuroImage Clin..

[73] Cipriano L., Minino R., Liparoti M., Polverino A., Romano A., Bonavita S., Pirozzi M.A., Quarantelli M., Jirsa V., Sorrentino G. (2024). Flexibility of brain dynamics is increased and predicts clinical impairment in relapsing-remitting but not in secondary progressive multiple sclerosis. Brain Commun..

[74] Romano A., Lopez E.T., Cipriano L., Liparoti M., Minino R., Polverino A., Cavaliere C., Aiello M., Granata C., Sorrentino G. (2023). Topological changes of fast large-scale brain dynamics in mild cognitive impairment predict early memory impairment: A resting-state, source reconstructed, magnetoencephalography study. Neurobiol. Aging.

[75] Polverino A., Troisi Lopez E., Liparoti M., Minino R., Romano A., Cipriano L., Trojsi F., Jirsa V., Sorrentino G., Sorrentino P. (2024). Altered spreading of fast aperiodic brain waves relates to disease duration in Amyotrophic Lateral Sclerosis. Clin. Neurophysiol..

[76] Nikulin V.V., Brismar T. (2004). Long-range temporal correlations in alpha and beta oscillations: Effect of arousal level and test-retest reliability. Clin. Neurophysiol..

[77] Klomjai W., Katz R., Lackmy-Vallée A. (2015). Basic principles of transcranial magnetic stimulation (TMS) and repetitive TMS (rTMS). Ann. Phys. Rehabil. Med..

[78] Berlim M.T., Van Den Eynde F. (2014). Repetitive transcranial magnetic stimulation over the dorsolateral prefrontal cortex for treating posttraumatic stress disorder: An exploratory meta-analysis of randomized, double-blind and sham-controlled trials. Can. J. Psychiatry.

[79] Edinoff A.N., Hegefeld T.L., Petersen M., Patterson J.C., Yossi C., Slizewski J., Osumi A., Cornett E.M., Kaye A., Kaye J.S. (2022). Transcranial Magnetic Stimulation for Post-Traumatic Stress Disorder. Front. Psychiatry.

[80] McGirr A., Devoe D.J., Raedler A., Debert C.T., Ismail Z., Berlim M.T. (2021). Repetitive Transcranial Magnetic Stimulation for the Treatment of Post-Traumatic Stress Disorder: A Systematic Review and Network Meta-analysis. Can. J. Psychiatry.

[81] Trevizol A.P., Barros M.D., Silva P.O., Osuch E., Cordeiro Q., Shiozawa P. (2016). Transcranial magnetic stimulation for posttraumatic stress disorder: An updated systematic review and meta-analysis. Trends Psychiatry Psychother..

